# The anticipation of imminent events is time-scale invariant

**DOI:** 10.1073/pnas.2518982123

**Published:** 2026-01-07

**Authors:** Matthias Grabenhorst, David Poeppel, Georgios Michalareas

**Affiliations:** ^a^Ernst Strüngmann Institute for Neuroscience in Cooperation with Max Planck Society, Frankfurt 60528, Germany; ^b^Department of Cognitive Neuropsychology, Max-Planck-Institute for Empirical Aesthetics, Frankfurt 60322, Germany; ^c^Department of Psychology, New York University, New York, NY 10003; ^d^CoBIC, Medical Faculty, Goethe University, Frankfurt 60323, Germany

**Keywords:** anticipation, prediction, scale invariance, time estimation, probability estimation

## Abstract

Humans predict the timing of imminent events to generate fast and precise actions, decisions, and other behaviors. Such temporal anticipation is critical over wide timescales, and especially salient over the range from hundreds of milliseconds to a few seconds. Despite advances in our understanding of basic timing behavior and its underlying neural mechanisms, it remains an open question whether anticipation is stable across these short time scales. Recent work shows that the brain models the probability density function (PDF) of events across time, suggesting a canonical mechanism for temporal anticipation. Here, we investigate whether this computation holds when the event distribution covers different time spans. We show that, irrespective of the time span, anticipation, measured as reaction time, scales with the event distribution. This demonstrates that the key computation—the estimation of event probability density—is invariant across temporal scales. We further show that the precision of anticipation is also scale invariant which contradicts Weber’s law. The results are established in vision and audition, suggesting that the core computations in temporal anticipation are independent of sensory modality. Perceptual systems exploit probability estimation over time independently of temporal scale to anticipate imminent events.

## Significance

From everyday conversation to sports, to traffic, to music, people constantly predict when events will happen so they can prepare their next actions. This study examines how the brain makes such timing predictions over short periods of a few seconds. Using experiments with vision and audition, along with models of reaction times, we found that people rely on the same underlying calculation regardless of the time scale: They estimate the probability of an event over time. This process drives anticipation and determines how precise anticipation is, consistently across time scales. Our results suggest that this scale invariance is a basic principle of how humans anticipate events in time—a core function that supports many aspects of thought and behavior.

The anticipation of future events shapes perception and cognition. In the range from hundreds of milliseconds to several seconds, anticipation influences many complex functions across different domains, including attention (1), decision-making (2, 3), motor control (4, 5), interpersonal synchronization (6) and interaction (7). At the subsecond level, imagine dancers quickly reacting to partners’ movements, gamers responding to sudden changes on-screen, or table tennis players engaged in a fast-paced rally. At the second scale, consider drivers braking in time to stop at a red light, or a bouncer at a club stepping in to prevent an imminent assault.

Successful anticipation requires estimation of the time point of a target event and the preparation of an action prior to this event. This mechanistically connects temporal anticipation to interval timing and motor timing.

**Fig. 1.**
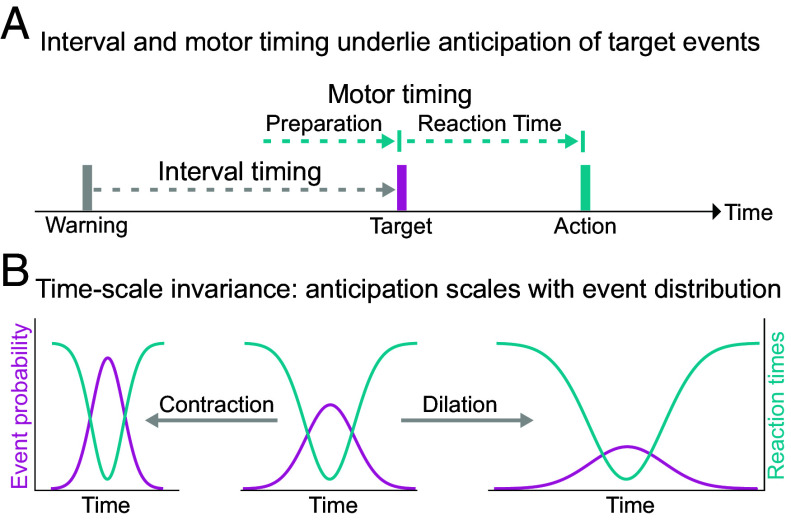
Hypotheses about temporal anticipation. (*A*) Target events follow warning events. Interval timing: time estimation between warning and target events. Motor timing: Based on prediction, a motor action is prepared before a target event occurs. After the target event is registered, the action is executed. Reaction time (RT) is the time between target event and motor action. (*B*) Temporal anticipation, measured as RT (y-axis *Right*; turquoise), is hypothesized to proportionally scale with target event probability (y-axis *Left*; purple) rescaled over time.

Interval timing is concerned with the estimation of time between two sensory events (8), e.g. a warning cue followed by a target cue (Fig. 1*A*). Such timing underlies timespan reproduction (9), interval discrimination (10), and sensorimotor synchronization (11, 12). Different neural mechanisms are thought to underpin interval timing, ranging from hypothesized cerebellar (8, 13) and cortico-striatal (8, 14) accounts to suggestions for timing as an intrinsic neural property (15), including subcellular (molecular) (16, 17) mechanisms.

Motor timing concerns the preparation and execution of a movement to an expected sensory cue (15), for example in the generation of a short reaction time (RT) (Fig. 1*A*). Several neural structures are involved over different time spans, including the basal ganglia on the scale of hundreds of milliseconds to seconds (15), the cerebellum in subsecond (18, 19) and potentially suprasecond (>2 s) (20) ranges, and cortical circuitry ranging from sensory to association to motor cortex (15), operating over sub-/perisecond (21, 22) to multiple-second (23, 24) durations.

In addition to evidence for distinct neural substrates supporting interval and motor timing, behavioral studies suggest that temporal processing operates across partially overlapping time scales rather than being differentially implemented across segregated scales. While some of the literature distinguishes between subsecond and suprasecond intervals (8, 25), this stance may reflect a convenient heuristic rather than a substantive boundary. Neural and behavioral findings indicate a graded transition between mechanisms around the one-second range, with overlapping contributions between approximately 0.5 and 1 s (26, 27). Some work proposes a shift in processes near 1.3 to 2 s (28–30), suggesting a continuum in which the relative engagement of mechanisms changes gradually with duration.

The results, on balance, favor scale-sensitivity in timing encompassing subsecond, second, and suprasecond intervals. Importantly, anticipatory behavior depends on accurate temporal prediction across this entire range. This raises the key question of whether anticipatory mechanisms themselves are tuned to specific temporal scales or operate according to scale-invariant principles.

Formally, scale invariance is a property of a system that remains unchanged under a rescaling of its input variables (31), i.e., the system’s output function retains the same shape but with a different scale. An anticipation system can be considered time-scale invariant if its predictive output scales with the distribution of events under temporal dilation or contraction (Fig. 1*B*).

In our environment, the distributions of many different features are scale invariant, i.e. the environment’s statistical structure is often constant over a change of scale (32, 33). This organizational principle affects biological agents within the environment and is reflected in scale-free brain dynamics and behavior (31). Notably, some human (34) and animal (35) timing behaviors, including basic interval timing (36–38), exhibit invariance over a wide range of temporal scales. Conditioning experiments suggest that interval timing between predictive cues and outcomes (39) and associative-learning itself (40) are time-scale invariant. Specifically, Gallistel and Gibbon’s Temporal Map Theory posits that organisms learn and represent the probability distribution of event timings in a scale-invariant manner, storing delays as ratios that preserve relative temporal structure across interval ranges (39). Whether this learning-centered framework extends to anticipation remains unknown and forms a central question of the present study. A further issue concerns whether the probability distribution of sensory events governs not only the mean but also the variance of anticipation, as recent evidence suggests that probability modulates temporal uncertainty (41), which contradicts classical scalar-noise models (36, 42).

Here we characterize how stimulus temporal statistics relate to human anticipatory behavior. We build on our recent work arguing that temporal anticipation is based on the estimation and representation of event probability over time (21, 41) and investigate whether this hypothesized core computation holds across different temporal scales.

## Results

**Fig. 2.**
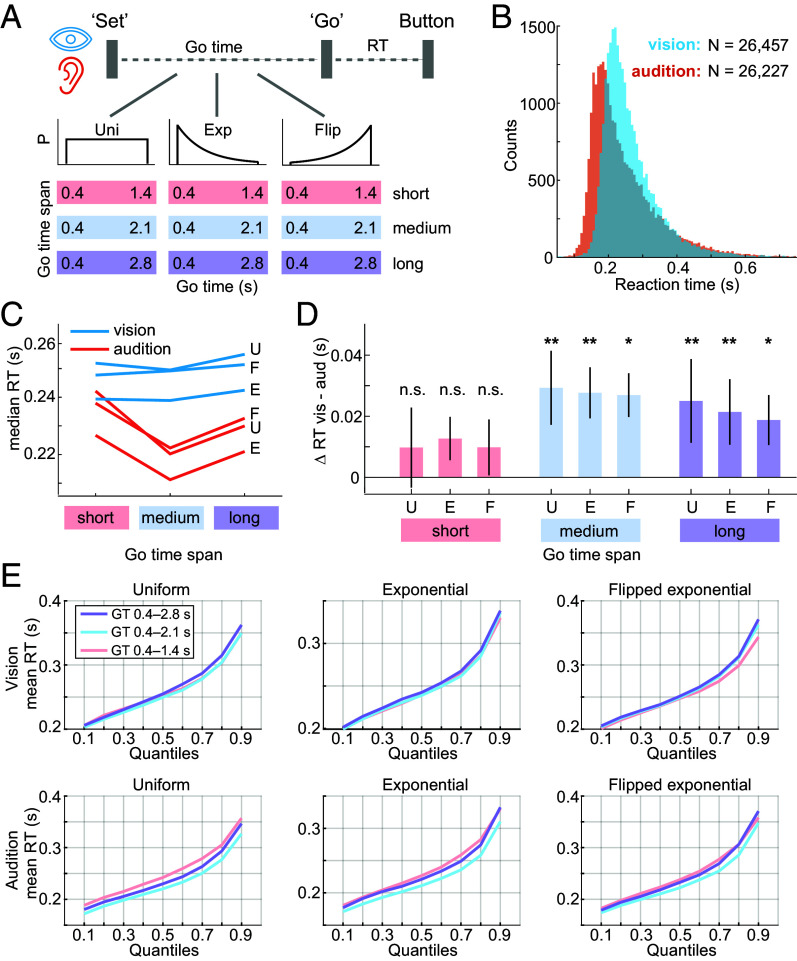
Task and basic analysis of RT. (*A*) Set-Go RT task. In visual and auditory blocks of trials, participants were asked to press a button as fast as possible in response to the Go cue, generating a RT. In 9.09 % of trials, no Go cue was presented. In this case, participants were asked to not press the button. In separate blocks of trials, the time between Set and Go (Go time) was drawn from truncated uniform (Uni), exponential (Exp), or flipped exponential (Flip) distributions. All distributions were defined over three different Go-time ranges. (*B*) Histograms of visual (blue) and auditory (red) RTs, pooled across the 13 participants and all experimental conditions. (*C*) Offset between visual and auditory average RT. Mean of median RT averaged across participants within short, medium, long Go-time spans in uniform (U), exponential (E), and flipped exponential (F) conditions. (*D*) Significant difference in average RT across vision and audition at medium and long Go-time spans. No significant difference at short Go-time span. Within each experimental condition, ∆ median RT was computed at the single-participant level across modalities (vision - audition) and then averaged (mean) at group level (**P < 0.01, *P < 0.05, two-tailed *t* test, *SI Appendix*, Table S1). All error bars are SEM. (*E*) Similarity of RT distributions across short, medium, and long Go-time conditions. Within participant and within experimental condition, RT quantiles were computed. The within-quantile mean RT was computed first at the single-participant level and then at the group level (Vincentizing) (43).

In both visual and auditory Set-Go tasks, a Set cue was followed by a Go cue (Fig. 2 *A*, *Top*). Participants were asked to press a button as fast as possible in response to the Go cue, generating a RT. The time between Set and Go, the Go time, was drawn from truncated uniform, exponential, and flipped exponential distributions. Each distribution was defined over three Go-time spans of different duration, ∆t = (1, 1.7, 2.4) s, each beginning at t = 0.4 s (Fig. 2 *A*, *Bottom*, *Materials and Methods*). This experimental design allows us to identify the effects of temporal duration on the key computations in event anticipation by relating stochastic sensory input to RT dynamics.

13 participants completed the experiment and generated 52,684 RTs. The distributions of visual and auditory RTs showed characteristic properties in line with the simple RT task: In both sensory modalities, the RT histograms had a steep left flank and a heavy right tail (Fig. 2*B*). 97.3% of visual RTs and 97.7% of auditory RTs fell inside of the interval of RT = (0.05, 0.75) s. Median RT was 20.8 ms shorter in audition (0.225 s, range: 0.165 to 0.356 s) than in vision (0.246 s, range: 0.207 to 0.315 s, *P* = 0.023, *t* (12) = −2.62, two-tailed paired *t* test). This across-modality difference in median RT was driven by medium and long Go-time conditions (Fig. 2*C*), where median RT differed significantly in each Go-time distribution condition (Fig. 2*D*). In the short Go-time conditions, median RT did not differ between sensory modalities. Across the three Go-time spans, the distribution of RT was very similar in each sensory modality and Go-time distribution condition (Fig. 2*E*), suggesting similarity in the underlying generative process.

**Fig. 3.**
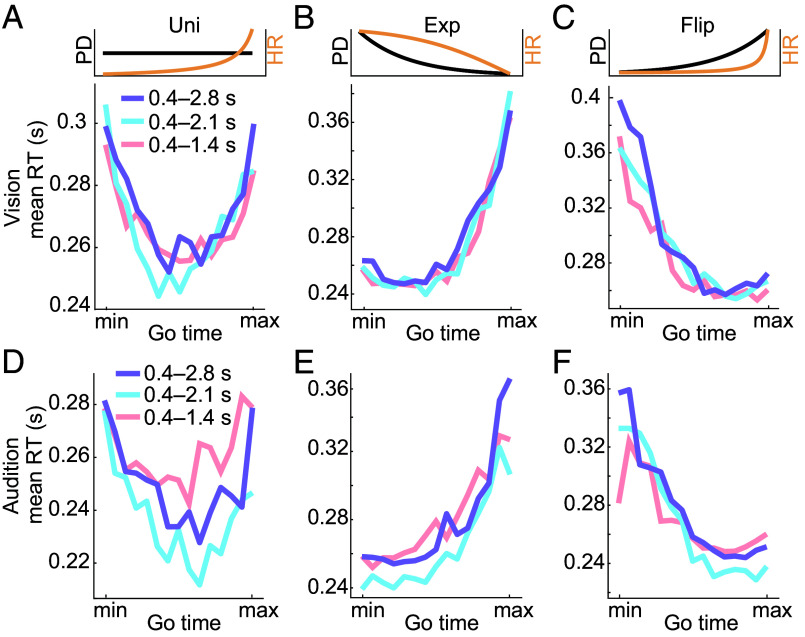
Anticipation is modulated by event distribution across time spans. Visual mean RT aggregated within each of the 15 individual Go times (*Materials and Methods*) in each of the three Go-time span conditions (short, medium, long) in (*A*) uniform, (*B*) exponential, and (*C*) flipped exponential conditions. Auditory mean RT aggregated within each of the 15 individual Go times in each of the three Go-time span conditions in (*D*) uniform, (*E*) exponential, and (*F*) flipped exponential conditions. All x-axes in arbitrary units. Inset figures depict uniform, exponential, and flipped exponential Go-time probability density (PD) functions (black) and corresponding hazard rates (HR, orange, N.b. computation of HR incorporates the catch trial percentage, *Materials and Methods*.) PDFs and HRs scaled to their respective ranges.

To investigate how the three Go-time distributions shape temporal anticipation, mean RT was plotted over Go-time. For easy reference, in each condition, we plotted the Go-time distributions as both probability density functions (PDFs) and their corresponding hazard rates (insets in Fig. 3 *A*–*C*). These variables are explained in detail in the modeling section below.

Across the three Go-time span conditions, the visual (Fig. 3 *A*–*C*) and auditory (Fig. 3 *D*–*F*) RTs were modulated by the Go-time distributions in a similar way. This similarity pertains to both the *shape* of the RT curves and the numerical *range* of the RT modulation. In the uniform case in vision, the RT curves approximated a U-shaped pattern (Fig. 3*A*). In audition, the U-shape in RT curves was qualitatively preserved (Fig. 3*D*) but there was a small, but nonsignificant, offset between the RT curves (one-way ANOVA on mean RT, *F_(2, 36)_* = 0.59, *P* = 0.56). In both exponential and flipped exponential conditions, the visual RTs were inversely related to event probability density: Where probability is high, RT is short and vice versa. Specifically, the two symmetric Go-time distributions (Exp and Flip) lead to close-to-symmetric patterns of visual RT (Fig. 3 *B* and *C*). Comparable RT dynamics were observed in the corresponding auditory conditions (Fig. 3 *E* and *F*). Importantly, the similarity of the RT dynamics across the three Go-time spans suggests similar neural computations based on the Go-time distributions. To investigate these computations, we next modeled the RT curves.

### Models of Anticipation Based On Probability Estimation Across Different Time Scales.

Recent results suggest that perceptual systems estimate the probability of sensory events over time (21, 41, 44). This information is summarized by the event probability density function (PDF), f(x), defined as $P\left(a\leq X\left(t\right)\leq b\right)=\int_{a}^{b}f_{X}\left(x,t\right)dx$, where *t* denotes time and P(a≤X(t)≤b) is the probability that the event X will happen in the time interval [*a*, *b*]. In modeling, an inverse relationship between probability-based anticipation and its behavioral correlate (RT) is realized by a nonlinear transformation, $\frac{1}{\mathrm{PDF}}$: where anticipation is strong, RT is short and vice versa (21, 41, 44).

**Fig. 4.**
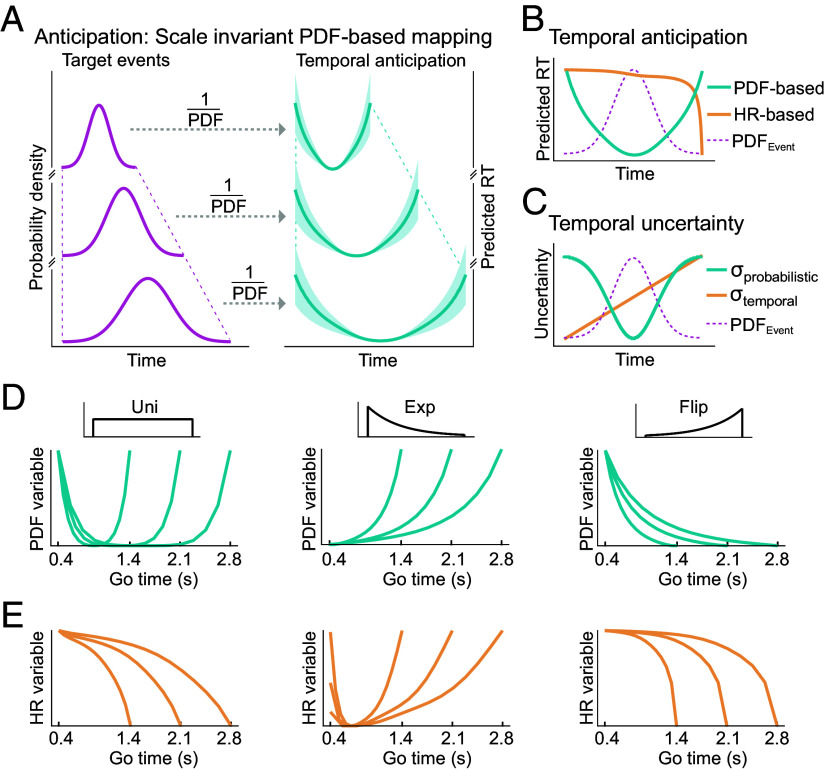
Hypotheses in temporal anticipation based on event PDF and hazard rate. (*A*) At different temporal scales, a neural prediction system is hypothesized to relate the probability of sensory events over time (*Left* curves) to anticipatory dynamics, measured as RT (*Right* curves), by computing the reciprocal event PDF, which here is illustrated as a Gaussian PDF. Irrespective of the Go-time span, the computation of reciprocal event PDF shapes anticipation (RT) and the precision in anticipation (variance of RT, shaded areas). (*B*) The dynamics of anticipation are hypothesized to be shaped by the reciprocal probabilistically blurred PDF (PDF-based model, *Materials and Methods*) or by the mirrored temporally blurred hazard rate (HR-based model, *Materials and Methods*). (*C*) The uncertainty in elapsed time estimation is hypothesized to scale linearly with the inverted event PDF (probabilistic blurring, *Materials and Methods*) or with elapsed time itself (temporal blurring, scalar property, *Materials and Methods*). (*D*) To-be-fit probabilistically blurred PDF variable in uniform, exponential, and flipped exponential cases, each computed over the three investigated Go-time ranges of t = (0.4, 1.4; 0.4, 2.1; 0.4, 2.8) s (denoted as “PDF variable” in plots, *Materials and Methods*). All PDF-based variables predict scale invariance of Go-cue anticipation over time. (*E*) Corresponding to-be-fit mirrored temporally blurred HR variable (denoted as “HR variable” in plots, *Materials and Methods*). (*A*–*E*) y-axes are in arbitrary units. For comparison, all variables were scaled by their respective ranges.

We hypothesize that the estimation of event probability over time, as formalized in the PDF-based model (41), is an elastic process that adapts to different temporal ranges over which the events occur. In other words, the brain is able to model the event PDF—whether it unfolds over short or long time spans—with a scale-independent mechanism (Fig. 4*A*).

The hazard rate (HR) model of anticipation (42, 45–51) provides an alternative set of hypotheses to the PDF-based model (Fig. 4*B*). The HR, h(t), represents the probability that an event is imminent, given that it has not yet occurred (52): $h\left(t\right)=\frac{f(t)}{1-F(t)}$, where $F\left(t\right)=\int_{-\infty }^{t}f\left(u\right)du$ is the cumulative distribution function (CDF). The inclusion of catch trials to the experiment ($P_{\mathrm{catch}}≅0.09$) lead to the CDF ceiling at $1-P_{\mathrm{catch}}$ (i.e. $c_{\max}≅0.91$ instead of $c_{\max}=1$ in the case of no catch trials). Thereby, the catch trial probability affects the shape of the HR (44). The inverse relationship between anticipation and RT is assumed to be linear in the canonical HR-based model (42, 45, 50). This is realized by mirroring the HR around a fixed value (*Materials and Methods*). Notably, the HR computation is implicated in the anticipation of the immediate future, i.e. events over short time spans in the range from approx. 0.3 s up to 3 s (42, 46–48, 50, 51).

In this imminent time horizon, the uncertainty in the estimation of elapsed time (since a warning cue, Fig. 1*A*) was shown to increase with the length of the time interval (36, 39), according to Weber’s law (*scalar property*). In modeling, we call this *temporal blurring* and it is realized by a Gaussian blurring kernel whose SD (sigma) linearly increases with elapsed time (*Materials and Methods*). The HR-based model incorporates temporal blurring (*Materials and Methods*) (42, 45, 50). Therefore, the model implies that temporal uncertainty is independent of the event probability density (Fig. 4*C*).

The PDF-based model questions the rigidity of the scalar property of time estimation in the presence of an event PDF. In such a case, it assumes that temporal uncertainty is significantly driven by the event PDF. This is called *probabilistic blurring* (41, 44): Where event probability is high, temporal estimates are precise and vice versa, i.e. the sigma of the Gaussian blurring kernel is inversely related to the Event PDF (Fig. 4*C*, *Materials and Methods*). Consequently, probabilistic blurring posits that the precision in estimating the time point of an imminent event is independent of the uncertainty of just estimating elapsed time itself, as proposed by Weber’s law.

Note that the uniform event PDF constitutes a special case: Event probability density is constant over Go time, leading to a fixed sigma of the Gaussian blurring kernel over Go time. This is different from the temporal blurring case, where the sigma linearly increases over Go time (*Materials and Methods*). Importantly, both blurring regimes incorporate the hypothesis that there is uncertainty about the exact temporal range of each Go-time distribution: Go times may be perceived as being shorter than the minimum Go time (t = 0.4 s) and longer than the maximum Go time [t = (1.4, 2.1, 2.8) s]. This hypothesis is reflected in all three conditions’ to-be-fit PDF and HR variables (*Materials and Methods*). In the uniform case, this leads to a U-shape of the reciprocal probabilistically blurred PDF variable (Fig. 4 *D*, *Left*, *Materials and Methods*).

To test which model better captures anticipatory behavior over the investigated Go-time spans, we fitted two linear models of RT. The explanatory variables used in the PDF- and HR-based models were different, namely the reciprocal probabilistically blurred PDF (Fig. 4*D*) and the mirrored temporally blurred HR (Fig. 4*E*, *Materials and Methods* and *SI Appendix*).

### Anticipation Based On Probability Density Estimation Is Time-Scale Invariant.

**Fig. 5.**
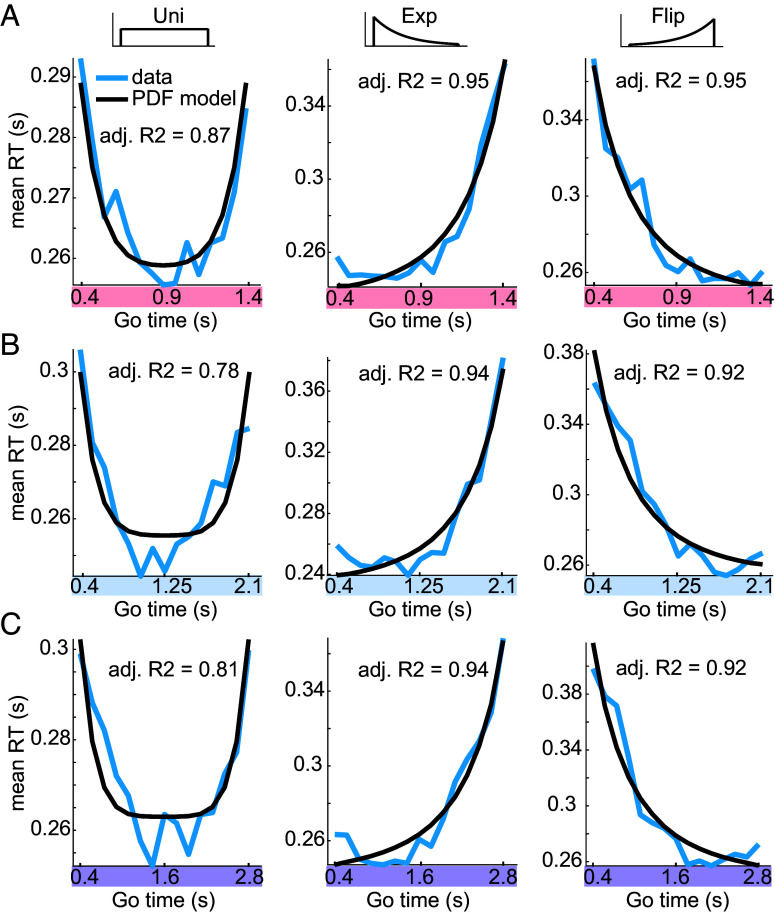
Estimation of event PDF is invariant across temporal scales. Fits to visual RT of the reciprocal, probabilistically blurred PDF (*Materials and Methods*). In all Go-time distribution conditions, the PDF-based model captures RT at (*A*) short, (*B*) medium, and (*C*) long Go-time spans.

The PDF-based (reciprocal, probabilistically blurred) and HR-based (mirrored, temporally blurred) explanatory variables were fit to group-level RT data using a linear model (*Materials and Methods*). The PDF-based model captured the RT dynamics well in all three Go-time distribution cases, in the short (Fig. 5*A*), medium (Fig. 5*B*), and long (Fig. 5*C*) time span conditions. These results also pertain to audition (*SI Appendix*, Fig. S1). Large values of adjusted R^2^ with small variance across experimental conditions support the PDF-based model fits in vision (adj. R^2^ = 0.90 ± 0.07, mean ± SD) and audition (adj. R^2^ = 0.77 ± 0.16, mean ± SD).

**Fig. 6.**
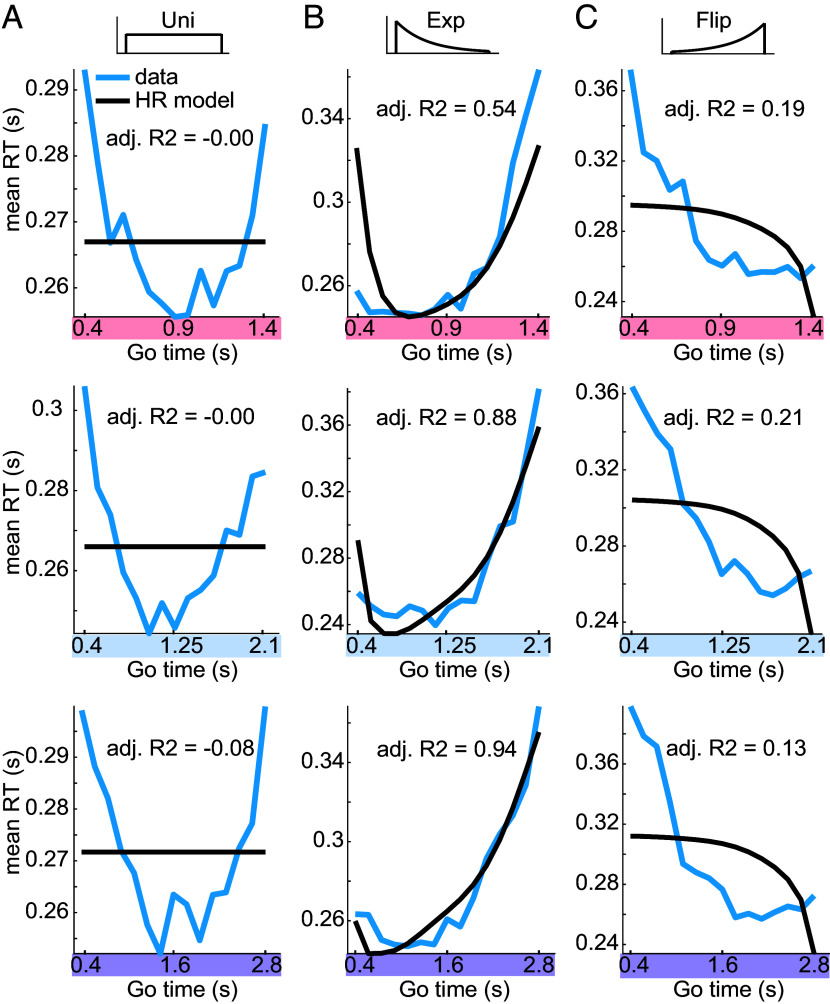
Hazard-Rate-based model does not capture anticipatory behavior across temporal scales. Fits of the mirrored, temporally blurred HR to visual RT (*Materials and Methods*). (*A*) In all uniform Go-time distribution conditions, the HR-based model fails to capture RT, even qualitatively (Fig. 4*E*). In fitting, the linear coefficient of the HR was effectively zero, resulting in a constant value of the HR model across Go time (*Materials and Methods*). (*B*) The goodness-of-fit of the HR-based model increases from short to long Go-time spans in the exponential Go-time distribution case. This was to be expected since both PDF-based and HR-based variables make qualitatively similar predictions in these conditions. (*C*) In all flipped exponential conditions, the HR-based model fails to capture the RTs.

The HR-based model failed to capture the RT modulation in all three Go-time span conditions in vision (Fig. 6) and audition (*SI Appendix*, Fig. S2). This is reflected in the substantially smaller values of adj. R^2^ (compared to the PDF-based model) in group-level fits in vision (adj. R^2^ = 0.31 ± 0.38, mean ± SD) and audition (adj. R^2^ = 0.28 ± 0.35, mean ± SD).

Two control analyses confirmed the results. First, we fit the PDF and HR models to single-participant data and compared adjusted R^2^ as a measure of goodness-of-fit at the group level. This single-participant analysis confirmed that the PDF model outperforms the HR model (*SI Appendix*, Figs. S3 and S4, Table S2). Second, in order to rule out that early guesses (motor initiation before Go-cue occurrence) affected our results, we removed all RT < 0.2 s before fitting the PDF-based models. This subselection led to an offset between RT curves while preserving similar shapes of RT curves over Go time (*SI Appendix*, Figs. S5 and S6) which were adequately fit by the PDF-based model (*SI Appendix*, Figs. S7 and S8).

Taken together, in each of the three investigated Go-time spans, the RT dynamics were well captured by the PDF-based model. This is consistent with the hypothesis that the neural estimation of event probability density, the key variable in anticipation, is invariant across temporal scales.

### The Precision of Anticipation Is Time-Scale Invariant.

**Fig. 7.**
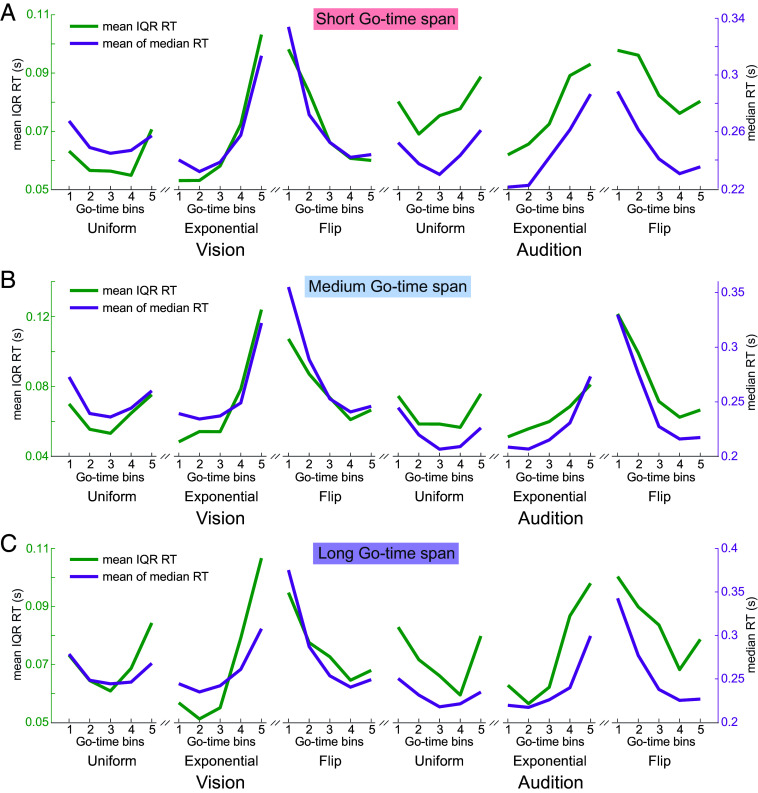
Anticipation and precision of anticipation are invariant across temporal scales. Interquartile range (IQR) of RT and median RT display a similar dynamic in all conditions at (*A*) short, (*B*) medium and (*C*) long Go-time spans. IQR of RT and median RT were computed in 5 bins, each consisting of 3 consecutive Go times, within-participant in each condition. Plots show group-level means per Go-time bin.

Next, we describe how the *precision* of temporal anticipation, quantified as RT variance, is affected by event probability over different Go-time spans. In all experimental conditions, within-participant, the interquartile range (IQR) of RT and median RT were computed, within 5 bins, each consisting of 3 consecutive Go times (Fig. 7, n.b. there were 15 individual Go times in each condition.).

To quantitatively investigate the precision of anticipation, we concatenated the IQR_RT_ values within each Go-time span condition (short, medium, long) and across distribution and sensory modality conditions, at the single-participant level. Median RT was concatenated in the same way. The concatenated IQR_RT_ vectors were averaged across participants within each Go-time span (short: IQR_RT_ = 0.074 ± 0.015 s, medium: IQR_RT_ = 0.071 ± 0.020s, long: IQR_RT_ = 0.074 ± 0.014 s). A one-way ANOVA revealed that there was no statistically significant difference in mean IQR_RT_ between any pair of Go-time span conditions [*F*(2, 27) = 0.29, *P* = 0.75]. This demonstrates that the precision of anticipation does not differ across the investigated time spans.

We next investigated how the dynamics over Go time of the precision of anticipation relate to each other across the different Go-time span conditions. At the single-participant level, Pearson correlation was computed on the IQR_RT_ vectors across all three combinations of Go-time span conditions. There was a significant positive correlation in all cases (Table 1). A one-way ANOVA on Pearson’s *r* revealed that there was no statistically significant difference in correlation between any pair of Go-time span conditions [*F*(2, 36) = 0.90, *P* = 0.41]. These results show that the dynamics of the precision of anticipation do not differ across the short, medium, and long Go-time spans.

**Table 1.** Pearson correlation computed at the single-participant level on IQR_RT_ between Go-time span conditions

Go-time span	Pearson’s *r(11)*	*P*	*t*(12)
short vs. medium	0.40 ± 0.22	0.00005	6.67
short vs. long	0.29 ± 0.20	0.0002	5.36
medium vs. long	0.38 ± 0.26	0.0002	5.28

*Note:* Paired-sample *t* test on Pearson’s *r* against zero, values are across-participant mean ± SD.

This led us to a final question: Across the three different time scales, how similar are the dynamics of RT to the dynamics of RT variance. Or, more intuitively, is the variance of RT modulated by event probability, in the same way average RT is? At the level of group averages, IQR_RT_ and median RT display a similar dynamic, in all experimental conditions and across short (Fig. 7*A*), medium (Fig. 7*B*), and long (Fig. 7*C*) Go-time spans. Indeed, in each Go-time span condition, IQR_RT_ and median RT were significantly positively correlated at the single-participant level (Table 2).

**Table 2.** Pearson correlation computed at the single-participant level between IQR_RT_ and median RT within Go-time span conditions

Go-time span	Pearson’s r(11)	*P*	*t*(12)
short	0.53 ± 0.12	1.3*10^−9^	16.51
medium	0.63 ± 0.14	1.2*10^−9^	16.67
long	0.43 ± 0.18	1.4*10^−6^	8.80

*Note:* Paired-sample *t* test on Pearson’s *r* against zero, values are across-participant mean ± SD.

Taken together, average RT and variance of RT share a linear relationship in their dynamics over all investigated temporal scales. This demonstrates that both the anticipation of sensory events over time and the precision of anticipation over time are jointly invariant across temporal scales.

This result is neither trivial nor intuitive. One would expect, according to the scalar property of time estimation, that the longer the Go-time spans, the larger the uncertainty in elapsed time estimation, leading to larger variance of RT. Moreover, this effect should be independent of probability. Here, we demonstrate that the precision of temporal estimation, captured by RT variance, is clearly modulated by probability and, accordingly, the RT variance can even decrease with time, as is seen here in the case of the flipped-exponential distribution (Fig. 7). This finding of “temporal precision modulation” by probability is observed to be identical in all three timespans, i.e., it is timescale-independent.

## Discussion

We investigated whether the anticipation of stochastic sensory events in the immediate future depends on the temporal scale of the event distribution or whether it is scale-independent. To this end, sensory events were distributed over three different time spans (short, medium, long) within the sub-three-second range, independently motivated by the existing literature.

Our data suggest that, in all three investigated timescales, observers estimate the event PDF to predict the timing of future events. This anticipatory mechanism shapes RT and the variance of RT in an identical way. We interpret this pattern as indicating that the estimation of event PDF by the brain is a time-scale invariant mechanism that drives both temporal anticipation and its precision. The hazard rate model, which has been implicated as a scale-invariant neural computation over the range of up to approximately three seconds (42, 46–48, 50, 51) and beyond (45, 53), in contrast, failed to capture the data patterns, irrespective of the time span.

Scale invariance offers several computational advantages in temporal anticipation. First, it suggests that time intervals are neurally represented in a relative fashion. This implies that the system does not have a preferred time scale (at least at the ranges we probed), which benefits flexibility in prediction (54). Second, a time-scale invariant prediction system may not need to learn different time-stretched versions of the same event distribution. Instead, the system may generalize its representations of temporal statistics across different contexts (31), promoting modularity and scalability across tasks. Third, this potentially reduces memory requirements and the amount of training needed to adapt behavior to changes in environmental temporal structure. For example, when a boxer faces a new opponent, similar temporal statistics will apply that were previously inferred from interaction with slower or faster opponents.

Scale invariance is conceptually related to fractality and supports hierarchical organization (31). In the neocortex, the time scale of neural integration gradually increases from sensory to association areas (55), realizing a temporal hierarchy in neural function (56). Time-scale invariant computation might accommodate the hierarchical processing of information, enabling prediction over different time scales, a feature of, e.g., human speech perception (57).

Our results have implications for the basic mechanisms of time estimation. The variability in human timing behavior is commonly thought to linearly increase with elapsed time itself (Weber’s law) (58). This so-called scalar property is often interpreted as time-scale invariance, e.g. in interval timing (36, 59). Our results do not support the scalar property/Weber’s law. Instead, across different time spans, RT and its variance were consistently shaped by the event PDF, but not by time itself. This surprising result demonstrates flexibility in the precision of temporal estimates and raises questions about its neural realization.

In our experiment, temporal dilation of the Go-time distribution—that is multiplying every interval by a constant factor—stretched the entire mean-RT and RT-variance curves rightward, in a similar way, while leaving their amplitudes largely unchanged. The neural mechanisms underlying these behavioral dynamics are currently unknown. In human posterior parietal and sensorimotor cortices, the event PDF is represented prior to anticipated events (21), but it is not clear whether this representation adapts to different time scales.

Recent theoretical and empirical developments converge on the idea that neural representations of time are inherently scalable. In the striatum, for example, a scalable population code was identified in which sequentially active neurons (“time cells”) dilate or contract their activity patterns proportionally with the interval being timed (60). Comparable temporal scaling was reported in cortical population dynamics during flexible timing behavior (61), and is also a key property of drift–diffusion models of interval timing, where rescaling of the accumulation process reproduces scalar variability in behavior (62). These observations suggest that temporally scalable neural architectures could implement the type of elastic probability distributions inferred from our data, offering a mechanistic link between behavioral invariance across time scales and underlying population-level dynamics.

Our behavioral results constrain the hypothesis space: Any such neural mechanism must contain a temporally scalable representation of the probabilistically blurred event PDF so that the form of the function relating Go time to RT has the same form across different Go-time ranges. One possible neural explanation behind these observations combines three computational elements.

(i) **Log-axis encoding.** Elapsed time may be represented on a logarithmically compressed timeline by a bank of leaky integrators (63). These can be pictured as a series of neural “hourglasses” whose activity fades at successively longer time-constants with a fixed scaling factor—e.g., 50 ms, 100 ms, 200 ms, 400 ms, and so on for a factor of two. Doubling every delay in a task (stretching an interval to double) simply doubles the moment at which each neuron peaks, so the whole activity pattern shifts later in time without changing its shape (64, 65). The brain could use the same fixed set of connections to read out elapsed time whether the intervals are short or long, giving the code its scale-invariance.
(ii) **Probability-based gain shaping.** As participants experience the task, the associated neural structures that carry each timing neuron’s signal to the movement-trigger neurons may adapt to match the learned event-time probability (39). When a go cue is likely, these synapses display strong connectivity; their combined input (“downstream drive”) pushes the movement-trigger neurons to threshold, and the reaction is fast. When the cue is unlikely, the synapses are weak; the drive builds more slowly, delaying the response. The summed drive therefore rises and falls in proportion to the probability density itself, producing the observed relationship RT ∝ 1/PDF(*t*) (66).
(iii) **Divisive normalization.** Our finding that RT variance is governed by probability rather than increasing with elapsed time implies the operation of a mechanism that counteracts the scalar property of timing (9). In a leaky-integrator model, longer intervals are represented more diffusely than shorter ones due to logarithmic compression: “early” neurons fire in narrow bursts, whereas “late” neurons fire in broader bursts whose width grows with latency. One way to narrow these late bursts and reduce their variance is divisive normalization, which scales down each neuron’s momentary activity by a slowly varying average of the entire population’s firing (67, 68). In practice, this normalization pool is larger when the PDF is high—because more neurons are driven strongly—resulting in a greater divisor at high-probability times and thus a lower effective gain of momentary activity. This suppression trims the flanks of broad late bursts, aligning their effective duration with early bursts and keeping the absolute variance—hence RT variance—constant, even as intervals lengthen (69).

This tentative framework aligns with Gallistel and Gibbon’s temporal-map theory, which predicts behavioral invariance under uniform scaling of all delays (39), and it extends that theory in two important respects. First, the present data suggest that temporal anticipation is governed by the learned probability density, not solely by delay ratios. Second, the observed constancy of absolute variance implies an additional variance-suppression stage absent from the original scalar-timing formulation. Collectively, the findings indicate that a learned probability distribution can, in principle, override scalar-time noise and become the principal factor determining both the accuracy and the precision of temporal anticipation.

Our results hold in both vision and audition, suggesting independence of sensory modality. Recent work provides evidence for a supramodal neural representation of the event PDF in the posterior parietal cortex (21). While tempting, one cannot argue that this representation also underlies time-scale invariant anticipation based on the present behavioral work alone. Conceptually, the impact of our experimental manipulations on the neural system can be investigated at different levels of analysis (70). Here, we investigate candidate computations at the algorithmic level but not their neural implementation. Consequently, we cannot answer whether the scale-invariant estimation of event PDF reflects a central representation or whether it reflects similar peripheral representations in the neural substrate of each sensory modality. This points to an important limitation of all behavioral work: Our conclusions are built on the implicit assumption that RT directly reflects the neural computations in the task. But in the absence of neural data, we cannot rule out that computations other than the estimation of the event PDF shape RT.

For example, the well-accepted central tendency effects may influence anticipation in addition to the event PDF. Such effects are observed in interval timing, e.g. in temporal (re-)production tasks (71–73), and predict shorter RT at the average foreperiod/Go-time (74–76). This may account for the RT modulation we observe in the uniform condition. Here, probabilistic blurring captures RT based on the uncertainty about the temporal range of the Go-time distribution. In the (flipped) exponential conditions, it is less clear how probabilistic blurring and event-PDF effects relate to the central-tendency effects introduced by the Go-time range. Future work needs to disentangle both effects. Still, at least in the uniform case, probabilistic blurring constitutes an alternative computational hypothesis to the central-tendency effects that relates anticipation to the uncertainty in time estimation. This differs conceptually from accounts based on optimality (9), or regression to the mean in a more general way (72).

In sum, our behavioral results show that humans estimate the PDF of imminent events—irrespective of the timescale over which they occur. This work adds to our understanding of scale-free computation in cognition.

## Materials and Methods

The experiments were approved by the Ethics Council of the Max-Planck Society. Written informed consent was given by all participants prior to the experiment.

### Participants.

A total of 16 healthy adults (7 female), aged 24 to 32 y, mean age 27.2 y, completed the three-day experiments. All were right-handed and had normal or corrected-to-normal vision, reported no hearing impairment and no history of neurological disorder. Participants received € 14 per hour. 3 participants were excluded from the analysis for lack of visual fixation (see below). 13 participants remained for analysis.

### Experimental Procedure.

Participants performed blocks of trials of visual and auditory Set-Go tasks. In the task, a SET cue was followed by a Go cue (Fig. 2*A* and *SI Appendix*). Participants were asked to respond as quickly as possible to the Go cue with a button press using their right index finger. Participants were instructed to foveate a central black fixation dot and restrict blinking to the timespan immediately following a button press. In 9.09% of trials, no Go cue was presented. In these catch trials, participants were asked not to press the button. This small percentage of catch trials was added to avoid possible strong effects of event certainty toward the end of the Go-time span (44). A small black circle around the central fixation dot was presented onscreen for 200 ms after a button press indicating the end of the trial. The intertrial interval (ITI) was defined by the onset of the small black circle and the Set cue of the following trial. The ITI was drawn randomly from a uniform distribution (range 1.4 to 2.4. s, discretized in steps of 200 ms). During the experiment, participants wore headphones and positioned their heads on a forehead-and-chin rest (Head Support Tower, SR Research Ltd.) at a fixed distance of approx. 60 cm relative to the computer monitor. An eye tracker (Eyelink DM-890, SR Research Ltd.) recorded participants’ eye movements at a sampling frequency of 500 Hz for fixation control. Trials in which visual fixation was not maintained within a radius of 2.5° visual angle around the central fixation point for more than 300 ms during the Go time were discarded for data analysis. Three participants who exceeded the cut-off of 7.5% of nonfixation trials were excluded from the analysis. 2.7% of visual RTs and 2.3% of auditory RTs fell outside of the range of RT = (0.05, 0.75) s and were discarded from analysis in order to exclude early guesses (RT < 0.05 s) and RT that are inappropriately long for a simple RT task (RT > 0.75 s) (52).

### Temporal Probabilities.

The time between Set and Go cues, the Go-time, was a random variable, drawn from either a uniform distribution (Eq. **1**), an exponential distribution (Eq. **2**) with parameter l=0.33 or from its left–right flipped counterpart (tau=3.0303 for both).

[1] $$f\left(t\right)=\frac{1}{b-a}for\;t\in [a,b],$$

[2] $$f\left(t\right)=\frac{1}{l}e^{-\left(\frac{t}{l}\right)}.$$

All Go-time distributions were delayed by 0.4 s and were defined over three different time spans t_1_ = (0.4, 1.4) s, t_2_ = (0.4, 2.1) s, t_3_ = (0.4, 2.8) s. Within each time span, each distribution was defined over 15 equally spaced individual Go-times. Sequential effects were reduced by the constraint that no more than two consecutive trials were allowed the same Go time. The Go-time distribution was fixed for a pair of consecutive blocks of trials. For each participant, the experiment composed of one session on each of three consecutive days. Each session consisted of six visual and six auditory blocks. A single block contained 132 trials of which 12 did not feature a Go cue (catch trials). The combinations of time span, Go-time distribution, and sensory modality were randomized across participants. Each combination of time span, Go-time distribution, and sensory modality was fixed for two consecutive experimental blocks of trials.

### Models of RT.

PDF-based and Hazard-rate-based models of RT were constructed to investigate the effect of the Go-time distribution on event anticipation. The presented uniform, exponential, and flipped exponential Go-time distributions are characterized by three functions, the event probability density function, the cumulative distribution function (CDF), and the hazard rate (HR):

$$\begin{array}{c} p(t_{\mathrm{go}}):PDF\;of\;RT\;as\;a\;function\;of\;Go\;time\;t_{\mathrm{go}}, \end{array}$$

[3] $$\begin{array}{c} c(t_{\mathrm{go}}):CDF\;of\;RT\;as\;a\;function\;of\;Go\;time\;t_{\mathrm{go}}=\int_{0}^{t_{\mathrm{go}}}p\left(u\right)du, \end{array}$$

[4] $$\begin{array}{c} h(t_{\mathrm{go}}):HR\;of\;RT\;as\;a\;function\;of\;Go\;time\;t_{\mathrm{go}}=\frac{p(t_{\mathrm{go}})}{1-c(t_{\mathrm{go}})}. \end{array}$$

### Mirrored Temporally Blurred Hazard Rate.

To arrive at the temporally blurred HR, each Go-time PDF was convolved with a Gaussian uncertainty kernel whose SD linearly increases with Go time relative to the Set cue: $\sigma =\varphi \cdot t_{\mathrm{go}}$. Here, $t_{\mathrm{go}}$ is the Go time and φ is a scale factor by which the SD σ of the Gaussian kernel increases, i.e. a Weber fraction for time estimation. We used a value of φ=0.21, consistent with previous research (30, 37, 41, 42, 44). The equations for the corresponding temporally blurred functions are

[5] $$p_{S}\left(t_{\mathrm{go}}\right)=\frac{1}{\varphi t_{\mathrm{go}}\sqrt{2\pi }}\underset{-\infty }{\int^{\infty }}p\left(\tau \right)\cdot e^{-{\left(\tau -t_{\mathrm{go}}\right)}^{2}/\left(2\varphi^{2}\tau^{2}\right)}d\tau ,$$

[6] $$c_{S}\left(t_{\mathrm{go}}\right)=\int_{0}^{t_{\mathrm{go}}}p_{S}\left(u\right)du,$$

[7] $$h_{S}\left(t_{\mathrm{go}}\right)=\frac{p_{S}(t_{\mathrm{go}})}{1-c_{S}(t_{\mathrm{go}})}.$$

For a given $t_{\mathrm{go}}$, the PDF is convolved with a Gaussian kernel centered at $t_{\mathrm{go}}$ (Eq. **5**). At $t_{\mathrm{go}}=0.4s$ after Set cue onset the kernel has SD φ·0.4. Similarly at $t_{\mathrm{go}}=\left[1.4,2.1,2.8\right]s$ the kernel has SD φ·[1.4,2.1,2.8]. In the computation of the subjective, temporally blurred PDF, the definition of the PDF was extended with zeros to the left and right of the Go-time range:

[8] $$\begin{array}{c} p_{\mathrm{padded}}\left(t_{\mathrm{go}}\right)\;=\;\left\{\begin{array}{c} 0,(0.4-3\cdot \varphi \cdot 0.4)\leq t_{\mathrm{go}}<0.4\\ p\left(t_{\mathrm{go}}\right),0.4\leq t_{\mathrm{go}}\leq 1.4\\ 0,[1.4,2.1,2.8]>t_{\mathrm{go}}\geq ([1.4,2.1,2.8]+3\cdot \varphi \cdot [1.4,2.1,2.8]) \end{array}\right.. \end{array}$$

The extensions were equal to three SD of the Gaussian kernel (encapsulating 99.7 % of the Gaussian uncertainty function) at the shortest and longest Go times. These extensions are necessary to capture the uncertainty that is introduced by under-/overestimation of minimum/maximum $t_{\mathrm{go}}$. Then the integral in Eq. **6** was computed between these new extrema [$\left(0.4-3\cdot \varphi \cdot 0.4\right)$, ([1.4,2.1,2.8]+3·φ·[1.4,2.1,2.8])] instead of the impractical interval of minus to plus infinity. For φ=0.21 the temporal range of the extended PDF (Eq. **8**) is also the range of integration in the computation of the subjective PDF in Eq. **5**. The PDF of each distribution, $p\left(t_{\mathrm{go}}\right)$ was normalized so that its integral from 0.4 s to *t_max_* s was 0.909 which reflects the 9.09% catch trials that did not feature a Go cue. Consequently, the maximum value of the CDF was 0.909. The HR was computed based on the PDF and CDF (Eq. **7**). To arrive at the to-be-fit HR variable, the HR was mirrored around its mean (Eq. **9**).

[9] $$\begin{array}{c} x_{\mathrm{mh}}\left(t_{\mathrm{go}}\right)=-\left(h\left(t_{\mathrm{go}}\right)-\bar{h}\right)+\bar{h}=-h\left(t_{\mathrm{go}}\right)+2\cdot \bar{h}\\ =-\frac{p\left(t_{\mathrm{go}}\right)}{1-c\left(t_{\mathrm{go}}\right)}+2\cdot \bar{h}, \end{array}$$

where

*x_mh_*: mirror of the hazard rate of the PDF

$\bar{h}$: meanHR.

### Reciprocal Probabilistically Blurred Event PDF.

Probabilistic blurring constitutes an alternative hypothesis to the temporal blurring described above. In probabilistic blurring, the uncertainty in elapsed time estimation depends on the event PDF: Go times with high probability of event occurrence are associated with low uncertainty in time estimation and vice versa, irrespective of the Go-time duration (41). In probabilistic blurring, the SD of the Gaussian kernel scales according to the event PDF. In order to keep the effects of temporal and probabilistic blurring comparable, we used the same range of values for the SD of the blurring Gaussian kernel for both: In the probabilistic blurring case, the minimum and maximum values were set according to the temporal blurring case as

[10] $$\sigma_{\min}=\varphi \cdot t_{\min}=\varphi \cdot 0.4\;\mathrm{and}\;\sigma_{\max}=\varphi \cdot t_{\max}=\varphi \cdot [1.4,2.1,2.8].$$

As in the HR case, the value of φ was likewise set to 0.21. The PDF under investigation was then scaled so that its minimum value is $\sigma_{\min}$ and its maximum value $\sigma_{\max}$.

If $p_{\min}$ and $p_{\max}$ are the minimum and maximum values respectively of the PDF under investigation then the function used for computing the SD of the Gaussian kernel based on the PDF $p\left(t_{\mathrm{go}}\right)$ was defined as

[11] $$\begin{array}{c} s\left(t_{\mathrm{go}}\right)=\left[1-\frac{\left(p\left(t_{\mathrm{go}}\right)-p_{\min}\right)}{\left(p_{\max}-p_{\min}\right)}\right]\cdot \;(\sigma_{\max}-\sigma_{\min})+\sigma_{\min}. \end{array}$$

The term inside the brackets demonstrates that when the probability $p\left(t_{\mathrm{go}}\right)$ is low the SD of the Gaussian kernel approaches $\sigma_{\max}$, while when the probability increases, $s\left(t_{\mathrm{go}}\right)$ approaches $\sigma_{\min}$.

The uniform event PDF constitutes a special case as its probability is fixed across Go time: $p_{t_{\mathrm{go}}}=p_{\min}=p_{\max}$, reducing Eq. **11** to the fixed value $s\left(t_{\mathrm{go}}\right)=\sigma_{\max}$ which results in a maximal blurring of the event PDF at each Go time. In order to keep the degree of blurring comparable across event PDF conditions, in the case of the uniform event PDF, we computed the SD of the Gaussian kernel as

[12] $$s_{\mathrm{uni}}\left(t_{\mathrm{go}}\right)=\frac{\sigma_{\min}+\sigma_{\max}}{2}.$$

Note that Eq. **12** implies that the Gaussian blurring kernel has a fixed SD σ across the entire Go-times range. This is in accord with the probabilistic blurring, where event probability (also fixed in the uniform case), but not time, determines temporal uncertainty. By extending all of the event PDFs before the blurring (zero padding; see above), the blurred PDF variables capture the hypothesized temporal uncertainty at the extrema of the Go-times range. In the uniform case, this results in the U-shaped variable (44); in the exponential and flipped exponential cases, this results in a shallower slope at the steep onset/offset of the event PDFs (41).

Based on Eq. **11** for exponential and flipped exponential cases and Eq. **12** for the uniform case, the SD of the Gaussian kernel was derived and the probabilistically blurred PDF $p_{p}\left(t_{\mathrm{go}}\right)$ was computed as:

[13] $$p_{p}\left(t_{\mathrm{go}}\right)=\frac{1}{s(t_{\mathrm{go}})\sqrt{2\pi }}\underset{-\infty }{\int^{\infty }}p\left(\tau \right)\cdot e^{-{\left(\tau -t_{\mathrm{go}}\right)}^{2}/\left(2s(t_{\mathrm{go}}{)}^{2}\right)}d\tau .$$

Finally, in order to implement the Gaussian blurring of Eq. **13** at the extrema of Go times, the definition of the PDF was extended to the left and right of the actual stimulus presentation interval by three SD of the corresponding smoothing Gaussian kernels, similar to the temporally blurred case described in Eq. **8**, as

[14] $$\begin{array}{c} p_{\mathrm{padded}}\left(t_{\mathrm{go}}\right)\;=\left\{\begin{array}{c} 0,(0.4-3\cdot s(0.4))\leq t_{\mathrm{go}}<0.4\\ p\left(t_{\mathrm{go}}\right),0.4\leq t_{\mathrm{go}}\leq 1.4\\ 0,[1.4,2.1,2.8]>t_{\mathrm{go}}\geq ([1.4,2.1,2.8]+3\cdot s([1.4,2.1,2.8])) \end{array}\right.. \end{array}$$

The extensions of the Go-times range depend on the SD function $s\left(t_{\mathrm{go}}\right)$, which itself depends on the PDF. To arrive at the to-be-fit PDF variable, the reciprocal of the probabilistically blurred PDF was computed: 1/PDF (Eq. **15**).

[15] $$x_{\mathrm{op}}\left(t_{\mathrm{go}}\right)=\frac{1}{p\left(t_{\mathrm{go}}\right)},$$

where

*x_op_*: ‘reciprocal’ PDF.

### Modeling RT with a Linear Model.

The mirrored, temporally blurred HR and the reciprocal, probabilistically blurred PDF variables were fit to RT data (aggregated within Go time within participants for single-participant fits, then averaged across participants for group-level fits) using a linear model. An Ordinary Least Squares (OLS) regression was employed for the computation of the regression coefficients using the MatLab *fit* function (The MathWorks, Natick MA, USA). Adjusted $R^{2}$ was used as a measure of goodness-of-fit for comparing the models’ relation to RT.

## Supplementary Material

Appendix 01 (PDF)

Dataset S01 (MAT)

## Data Availability

Reaction times data have been deposited in Edmond—the Open Research Data Repository of the Max Planck Society (https://doi.org/10.17617/3.MEAGMS) (77).
