# Supplementary material for: The anticipation of imminent events is time-scale invariant
Source: Proc Natl Acad Sci U S A. 2026 Jan 7;123(2):e2518982123. doi: 10.1073/pnas.2518982123 (PMC12799109; doi:10.1073/pnas.2518982123)
Supplement: Supplementary file 1 — Appendix 01 (PDF) [file pnas.2518982123.sapp.pdf]

PNAS

Supporting Information for

The anticipation of imminent events is time-scale invariant

Matthias Grabenhorst*, David Poeppel, Georgios Michalareas

Corresponding author: Matthias Grabenhorst
Email: m.g@ae.mpg.de

This PDF file includes:

Supporting text
Figures S1 to S8
Tables S1 to S2
Legend for Dataset S1 (data_2025_18982.mat)
SI References

Other supporting materials for this manuscript include the following:

Dataset S1 (data_2025_18982.mat)

Supporting Information Text

Results

Hazard Rate. In the uniform conditions, the HR-based variable predicts decreasing RT over time (Fig. 4e, left). Since the RT curves are U-shaped, the fitting algorithm collapsed the HR-based model to a horizontal fit line close to the mean RT (Fig. 6a, Fig. S2a). The fits of the HR-based model are more adequate in the exponential condition, approaching the PDF-based models' goodness-of-fit (adj. R^2) (compare Fig. 5, middle, and Fig. 6, middle, Fig S1, middle, and Fig. S3, middle). This was to be expected since both PDF and HR variables make qualitatively similar predictions in the exponential case at medium and long Go-time spans (Figs. 4d middle, 4e middle). In the flipped exponential conditions, the HR-based model makes the qualitatively wrong prediction of a convex RT curve, where the RT curve is concave (Fig. 6c, Fig. S2c). A convincing model of anticipation needs to fit RT in all experimental conditions and the HR-based model fails to do so.

Methods

Visual stimuli. The Set cue consisted of two checkerboard patterns, which were presented simultaneously. One was positioned to the left of a central black fixation dot and the other on the opposite side. The Go cue consisted of two checkerboard patterns at the same location but with the black–white pattern reversed. Each checkerboard subtended 6.5 × 6.5° of visual angle and consisted of 7 × 7 black and white squares of equal size. The center of each checkerboard was positioned at a horizontal distance of 8.7° of visual angle and at a vertical distance of 0° from the center of the central fixation dot. Visual stimuli were generated using MatLab version 2023a (The MathWorks, Natick, MA, USA) and the Psychophysics Toolbox version 3(1). Set and Go stimuli were each presented for 50 ms on a BenQ XL2420-B monitor (resolution 1,920 × 1,080, refresh rate 120 Hz), which was set to a gray background.

Auditory stimuli. Two white noise bursts (50 ms duration, 8 ms cosine ramp, onset and offset) served as Set and Go cues. Auditory stimuli were generated using MatLab version 2023a (The MathWorks, Natick, MA, USA) and the Psychophysics Toolbox version 3(1). The stimuli were presented diotically at the same volume level for all subjects (~60 dB SPL) using an RME Fireface UCX interface and electrodynamic headphones (Beyerdynamic DT 770 PRO) driven by a headphone amp (Lake People GT-109).

PDF and HR variables. The to-be-fit PDF-based and HR-based variables differ in their predictions in the uniform and flipped-exponential cases, whereas in the exponential cases their predictions are qualitatively similar, i.e., both variables numerically increase over most of the Go-time range. Note that in the uniform case (Fig. 4e left), the temporally blurred variable decreases with Go-time while the probabilistically blurred PDF variable exhibits a symmetrical U-shape (Fig. 4d left). This difference results from the hypothesized increase in temporal uncertainty over Go-time in the temporal blurring case. Note that both blurring regimes hypothesize temporal uncertainty at the extrema of the Go-times range, which in the case of probabilistic blurring results in the U-shaped variables (Methods).

Supplementary Tables & Figures

Table S1. Two-tailed t-test on median RT across vision and audition.

**Go-time span**	**Distribution**	***p***	***t*(12)**
short	uni	0.47	-0.74
short	exp	0.32	-1.05
short	flip	0.49	-0.72
medium	uni	0.001	-4.15
medium	exp	0.006	-3.31
medium	flip	0.027	-2.52
long	uni	0.018	-2.74
long	exp	0.011	-3.00
long	flip	0.040	-2.30

Note: all P values uncorrected.

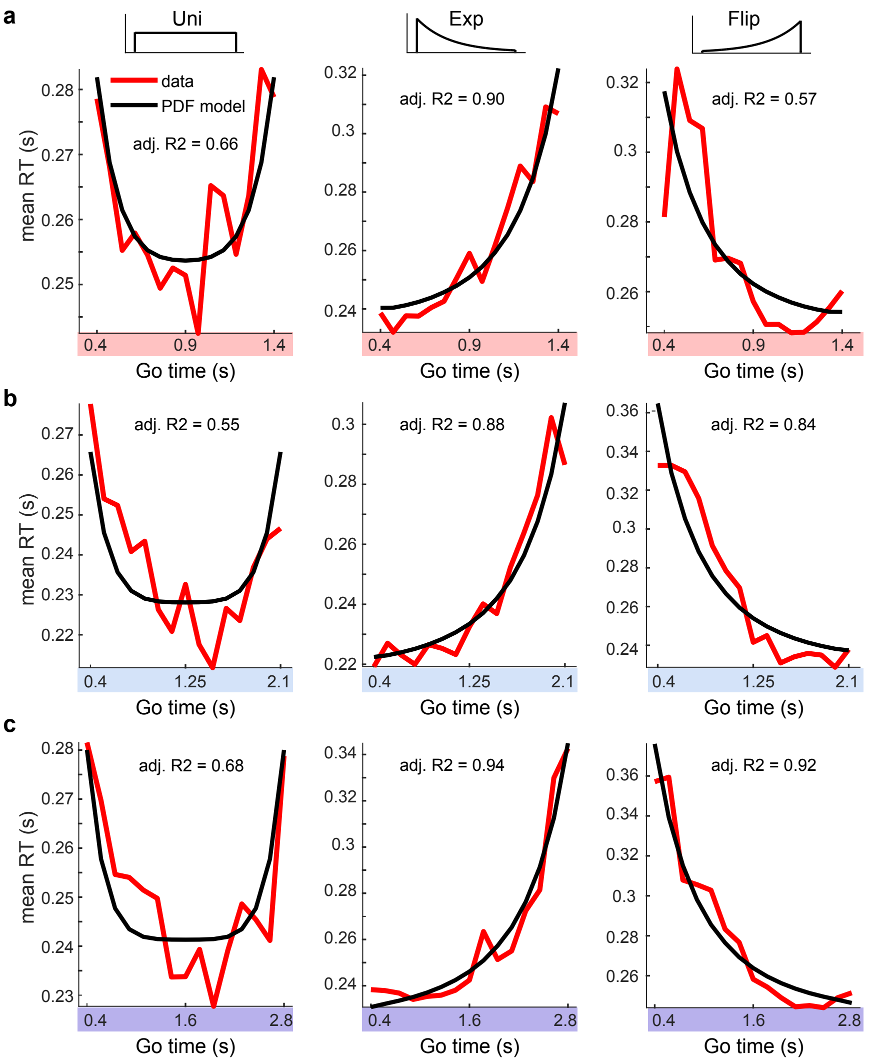

Fig. S1. Estimation of event probability density is invariant across temporal scales driving anticipation. Fits to auditory RT of the reciprocal, probabilistically blurred PDF (Methods). In all Go-time distribution conditions, the PDF-based model captures RT at **a**) short , **b**) medium, and **c**) long Go-time spans.

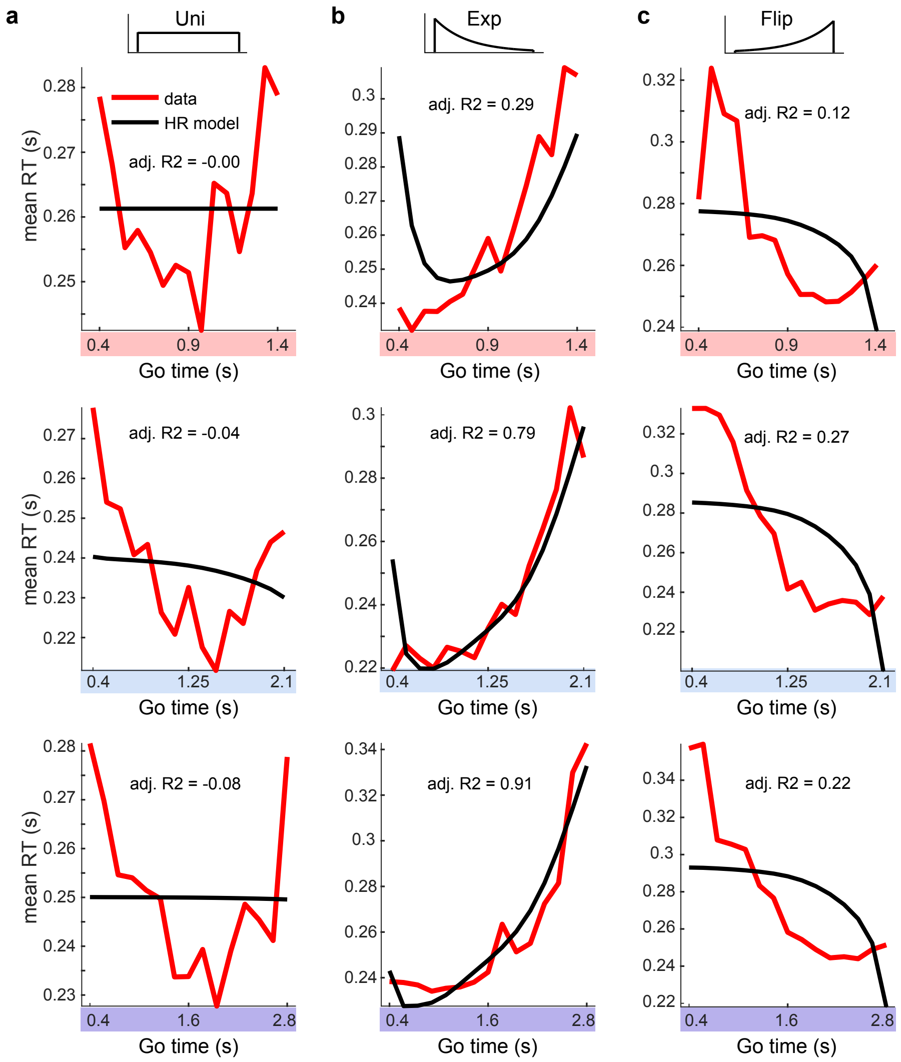

Fig. S2. Mirrored, temporally blurred hazard rate model fails to capture anticipatory behavior. Fits to auditory RT of the mirrored, temporally blurred HR (Methods). **a**) In all uniform Go-time distribution conditions, the HR-based model fails to capture RT. **b**) The goodness-of-fit of the HR-based model increases from short to long Go-time spans in the exponential Go-time distribution case. This was to be expected since both PDF-based and HR-based variables make qualitatively similar predictions of RT in these conditions (See Fig. 4d and e). **c**) In all flipped exponential conditions, the HR-based model fails to capture the RTs.

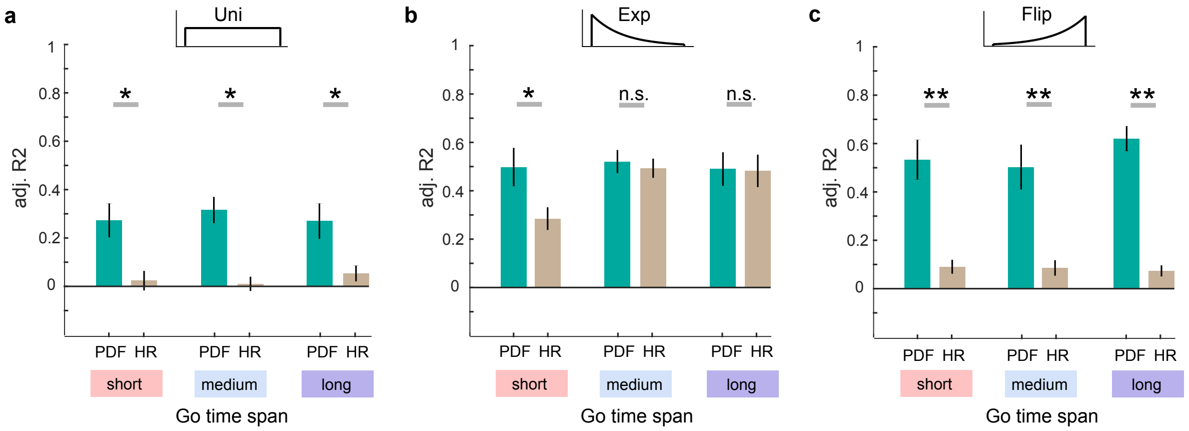

Fig. S3. PDF-based model captures single-participant RT in all three Go-time spans outperforming HR-based models. Plots of adj. R^2 from single-participant fits to visual RT of the PDF-based model for the three Go-time spans. The adj. R^2 for fits of HR-based models are also plotted for confirming the results from the group analysis. In general, the adj. R^2 values were smaller than in the group analysis, which is to be expected due to variability across participants. The significantly better fit of the PDF-based model compared to the HR-based one was also confirmed here. The statistical significance of this difference is represented by asterisks in each corresponding case. **a**) In the uniform Go-time distribution condition, as in the group analysis, the models had the smallest adj. R^2. This value was consistent across the three Go-time spans and adj. R^2 was significantly larger for all PDF-based compared to the HR-based models. **b**) In the exponential Go-time distribution condition, across all three Go-time spans, the PDF-based model performed well, with overall large adj. R^2 values. The HR-based model performed also well, with the only significantly smaller adj. R^2 in the short GT-span condition. **c**) In all flipped exponential Go-time distribution cases, the PDF-based model yielded large adj. R^2 values at the single-participant level in all three Go-time spans. Here the significant difference in adj. R^2 to the HR-based model was largest. (** $P < 0.01$, * $P < 0.05$, two-tailed t-test, Suppl. Table 2). Error bars are standard error of the mean.

Table S2. Two-tailed t-test on adj. R^2 across PDF-based and HR-based model fits to single-participant reaction times. Two-tailed t-test computed within-modality (vision and audition) and within Go-time span and distribution conditions.

Go-time span	Distribution	Vision		Audition	
		p	***t*(12)**	***p***	***t*(12)**
short	uni	0.0215	2.64	0.13	1.61
medium	uni	0.0018	4.00	0.012	2.97
long	uni	0.048	2.20	0.005	3.45
short	exp	0.004	3.57	0.003	3.66
medium	exp	0.174	1.44	0.094	1.82
long	exp	0.586	0.56	0.25	1.22
short	flip	$2 * 10^{-5}$	6.75	0.027	2.52
medium	flip	0.0002	5.34	$4 * 10^{-4}$	5.06
long	flip	$8 * 10^{-8}$	11.82	0.0017	4.02

Note: all P values uncorrected.

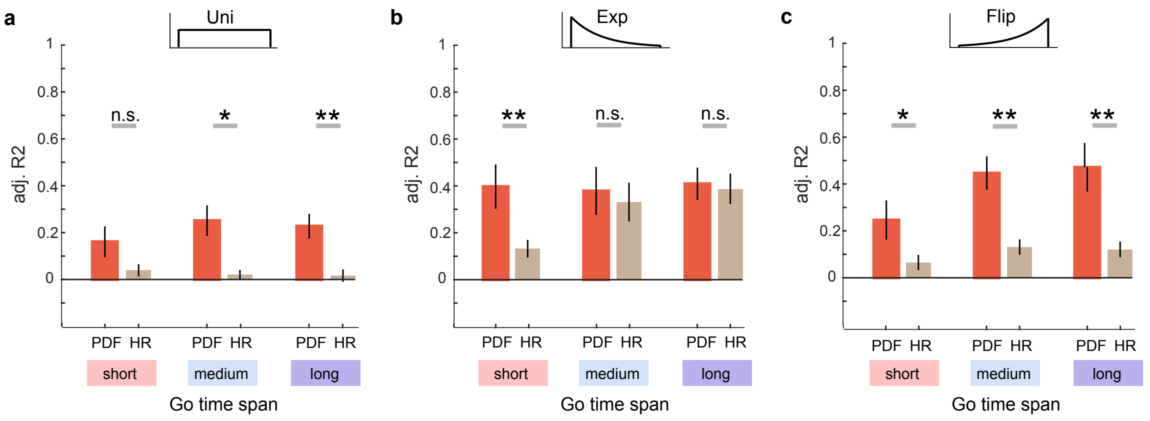

Fig. S4. PDF-based model outperforms HR-based model in single-participant fits to auditory RT. Comparison of adj. R^2 from single-participant fits of the reciprocal probabilistically blurred PDF and the mirrored temporally blurred HR (Methods). **a**) In all uniform Go-time distribution conditions, the PDF-based model yielded significantly larger values of adj. R^2 than the HR-based model. **b**) In the exponential Go-time distribution conditions, the goodness-of-fit between the PDF-based and the HR-based models differs at the short GT-time span but not at medium and long Go-time spans. This was to be expected since both PDF-based and HR-based variables make qualitatively similar predictions of RT in these conditions (See Fig. 4d and e). **c**) In all flipped exponential Go-time distribution conditions, the PDF-based model yielded significantly larger values of adj. R^2 than the HR-based model. (** $P < 0.01$, * $P < 0.05$, two-tailed *t*-test, Suppl. Table 2). Error bars are standard error of the mean.

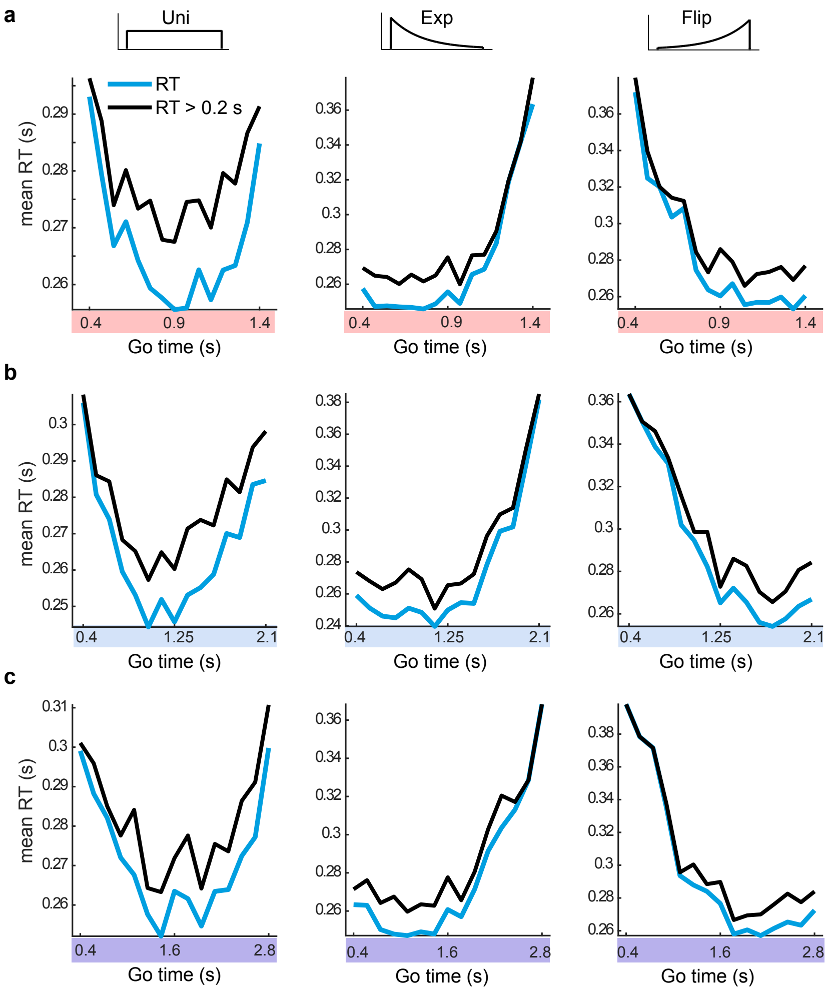

Fig. S5. Comparison of RT dynamics between different sub-selections of visual RT. Mean RT plotted over Go-time. Black curves show a sub-selection of RT = [0.2, 0.75] s (N = 21716 RTs). Blue curves show RT = [0.05, 0.75] s (N = 26457 RTs) in **a**) short , **b**) medium, and **c**) long Go-time spans. In all conditions, the sub-selection of RT led to an offset between curves while RT shapes remain very similar.

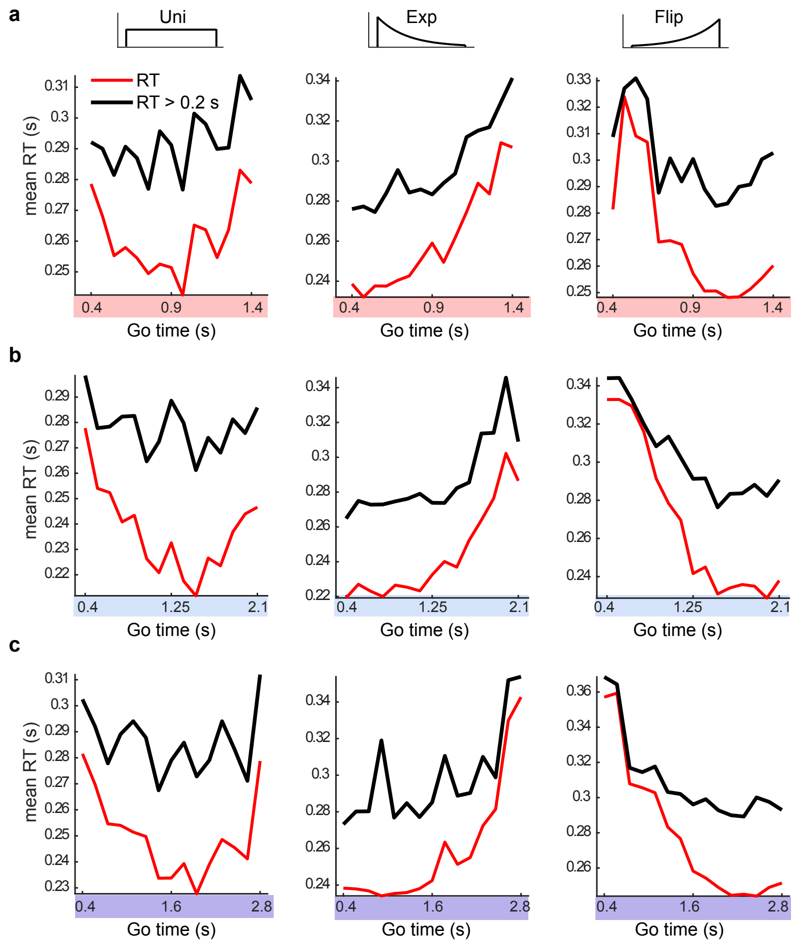

Fig. S6. Comparison of RT dynamics between different sub-selections of auditory RT. Mean RT plotted over Go-time. Black curves show a sub-selection of RT = [0.2, 0.75] s (N = 15476 RTs). Red curves show RT = [0.05, 0.75] s (N = 26227 RTs) in **a**) short , **b**) medium, and **c**) long Go-time spans. In all conditions, the sub-selection of RT led to an offset between curves while RT shapes remain similar.

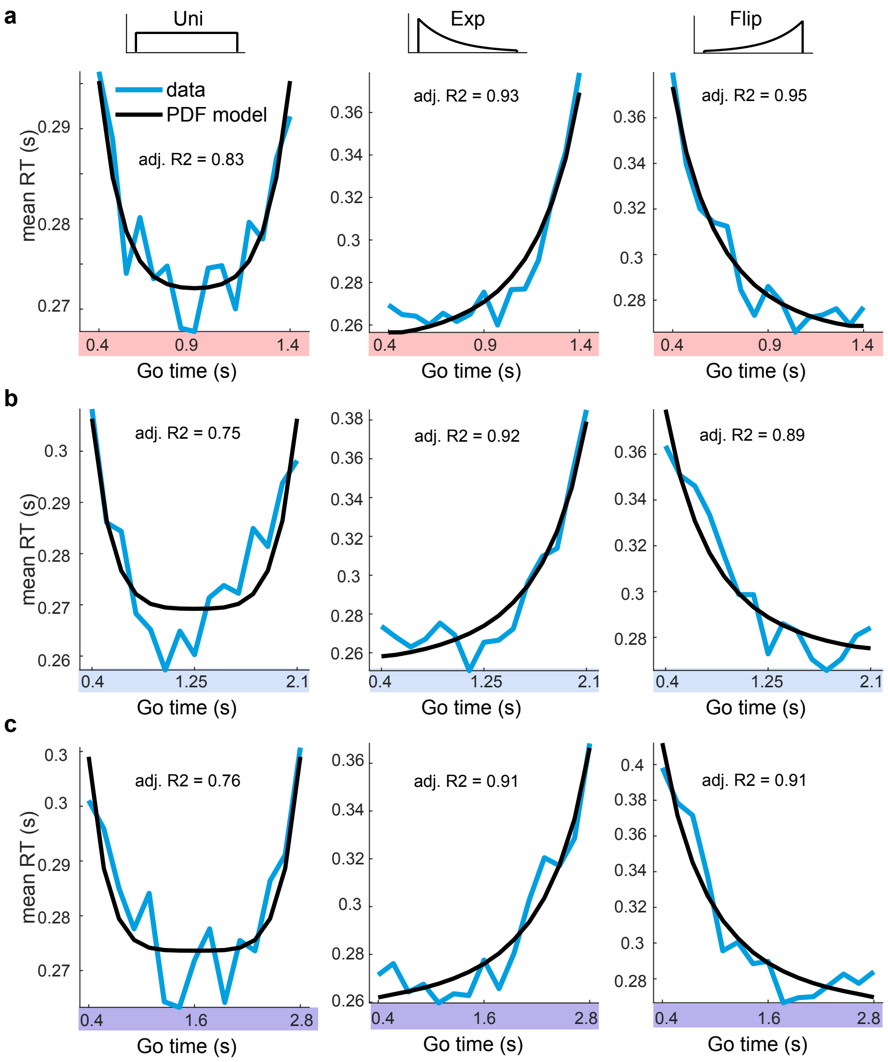

Fig. S7. PDF model captures RT dynamics of sub-selected visual RT. Fits of the reciprocal probabilistically blurred PDF (Methods) to a sub-selection of visual RT = [0.2, 0.75] s. In all Go-time distribution conditions, the PDF-based model captures RT at **a**) short , **b**) medium, and **c**) long Go-time spans.

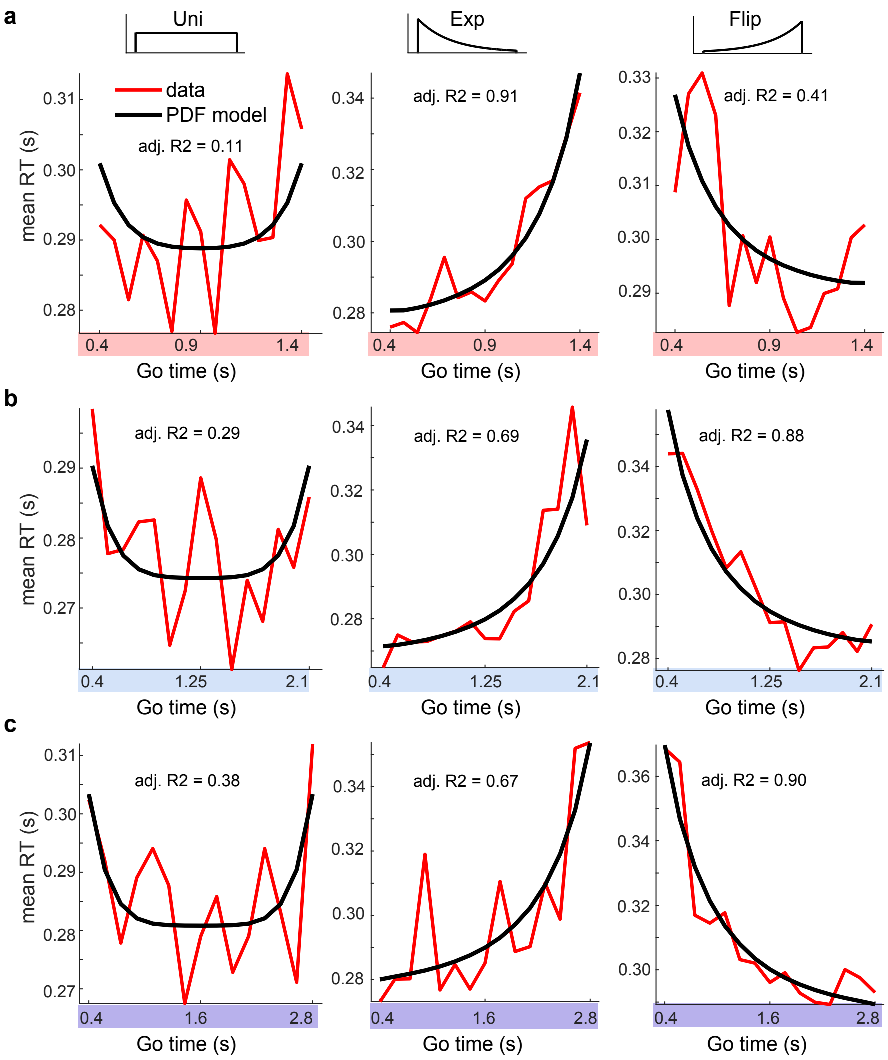

Fig. S8. PDF model captures RT dynamics of sub-selected auditory RT. Fits of the reciprocal probabilistically blurred PDF (Methods) to a sub-selection of auditory RT = [0.2, 0.75] s. In all Go-time distribution conditions, the PDF-based model captures RT at **a**) short , **b**) medium, and **c**) long Go-time spans.

Legend for Dataset data_2025_18982.mat
The reaction time data ('RTGTMat') are organized as a 5-dimensional matrix that has this structure: 13 participants x 15 Go times x 3 time spans (short, medium, long) x 3 Go time distribution conditions (uniform, exponential, flipped exponential) x 2 sensory modalities (audition, vision). The data comprise mean RT, averaged within-participant, within Go time.

References

1. D. H. Brainard, The Psychophysics Toolbox. *Spatial vision* **10**, 433-436 (1997).
